# Reproductive Ecology of the Invasive Alien Shrub *Pyracantha angustifolia* in the Grassland Biome, South Africa

**DOI:** 10.3390/plants12061308

**Published:** 2023-03-14

**Authors:** Lehlohonolo D. Adams, Dino Giovannoni, Vincent R. Clark, Sandy-Lynn Steenhuisen, Grant D. Martin

**Affiliations:** 1 South African National Biodiversity Institute, Centre for Functional Biodiversity, School of Life Sciences, University of KwaZulu-Natal, Pietermaritzburg 3209, South Africa; 2 Afromontane Research Unit & Department of Plant Sciences, University of the Free State, Phuthaditjhaba 9866, South Africa; 3 Centre for Invasion Biology, Department of Plant Sciences, University of the Free State, Phuthaditjhaba 9866, South Africa; 4 Department of Physics, Rhodes University, Makhanda (Grahamstown) 6139, South Africa; 5 Afromontane Research Unit & Department of Geography, University of the Free State, Phuthaditjhaba 9866, South Africa; 6 Afromontane Research Unit & Department of Zoology and Entomology, University of the Free State, Phuthaditjhaba 9866, South Africa; 7 Centre for Biological Control, Department of Entomology and Zoology, Rhodes University, Makhanda (Grahamstown) 6139, South Africa

**Keywords:** non-native shrub, seed biology, austral temperate grasslands, plant reproduction, insect pollination

## Abstract

Knowledge on reproductive traits of problematic invasive alien plants, such as the woody invasive shrub *Pyracantha angustifolia* of temperate Chinese origin, can help better manage invasive species. To determine factors contributing to its invasion, we investigated floral visitors and pollen loads, self-compatibility, seed set, seed rain, soil seed banks, and seed longevity in the soil. Generalist insects were recorded visiting flowers and all carried pollen loads of high purity (>70%). Floral visitor exclusion experiments showed that *P. angustifolia* can set seed (66%) without pollen vectors, although natural pollination resulted in higher fruit set (91%). Fruit count surveys and seed set showed an exponentially increased relationship between seed set and plant size with high natural seed yield (±2 million seeds m^−2^). Soil core samples revealed a high seed density of 46,400 ± (SE) 8934 m^−2^ under shrubs, decreasing with distance away from the shrub. Bowl traps stationed under trees and fences confirmed that seeds were efficiently dispersed by animals. Buried seeds survived for less than six months in the soil. Due to high seed production, self-compatibility augmented by generalist pollen vectors, and effective seed dispersal by local frugivores, it is difficult to manage the spread manually. Management of this species should focus on the short life span of seeds.

## 1. Introduction

Invasive alien plants (IAPs) are an important challenge to indigenous biodiversity management and safeguarding ecosystem services [1]. Understanding invasive plant reproductive traits is an effective information tool that is used to assist in their management [2,3], as most plant functional traits linked to invasion ability, are associated with reproduction [2,4]. Reproductive ecology covers all aspects of reproductive events, and their interactions with biotic and abiotic components of the environment [5]. These include fruiting and flowering phenologies [6,7], pollination [8,9], seed dispersal [10], seed density [11], and germination [12].

In southern Africa, invasive Rosaceae species of northern temperate origin are particularly striking in temperate and montane grasslands due to their red fruits being highly visible against a brown winter landscape. Invasive genera in this guild which have invaded southern African grasslands include *Cotoneaster* Medik. (several species), *Rubus* L. (several species), *Pyracantha* M. Roem. (several species), and *Rosa* L. (*Rosa rubiginosa*)—all introduced through horticultural origins. These species often form complex Rosaceae thickets, invading rangelands, cliff-lines, and watercourses [13]. These woody invaders, together with those that are not red-berried, pose a serious threat to high elevation grassland ecosystems because they are large compared to native grasses and thus transform the vegetation structure [1]. For example, shifts in native grass communities to shade tolerant invasive grass species were observed after invasion by *Robinia pseudoacacia* L. (Fabaceae) [13,14]. The high elevation grasslands of South Africa are key biodiversity hotspots with high plant endemism and provide half of the country’s water run-off [9,15]. Therefore, these IAPs threaten important ecosystem services provided by the grassland biome [16]. The biome is very valuable to the economy [16,17].

*Pyracantha angustifolia* (Franch.) C. K. Schneid. started invading the grassland biome of South Africa in the early 1980s [18]. However anecdotal evidence suggests it may have been planted as early as 1908 in Ladybrand in the Free State by a British pharmacist who made and sold high vitamin C juice from *Pyracantha* fruits (LD Adams, unpublished data). It is now widespread in the temperate grasslands of the Eastern Cape, Free State, KwaZulu–Natal, and Mpumalanga provinces, as well as in the adjacent countries of Lesotho, Eswatini, and Zimbabwe [19,20]. *Pyracantha angustifolia* has also naturalised in Argentina [21]; Australia [22]; Brazil [23]; Canada, Columbia, England, and France [24]; the French Polynesian Islands and Hawaii [25]; Italy, Japan, Mexico, New Zealand, Portugal, and Russia [25]; Spain, and the United States of America [26,27]; and Germany [28]. In southern Africa, by transforming an open habitat into a “*Pyracantha* savannah” or “thicket”, *P. angustifolia* competes with and displaces native plant species, erodes habitat suitability for endemic grassland fauna (many being of high conservation concern), provides a nursery habitat for additional invasive species, alters fire regimes, reduces rangeland capacity and dependent livelihoods, and reduces eco-tourism revenue when invading places of scenic beauty [13,29]. Due to the impact of the species, it is listed as a category 1b invasive species under the National Environmental Management: Biodiversity Act 963 (NEMBA, Act 10 of 2004) Alien and Invasive Species Regulations [30]. This act prohibits the importation, propagation, and trading of *P. angustifolia* in South Africa and requires it to be managed through the development and implementation of a management plan.

Empirical data published on *P. angustifolia* includes distribution and occurrence [13,15,31,32,33,34,35,36], germination [22,37], plant recruitment [38,39,40], uses by humans [41], impact on frugivore populations in association with *P. angustifolia* fruit abundance [42,43,44], and seed viability after ingestion by mammals and birds [37,45]. Although soil seed banks have been investigated, soil seed bank viability and distance from the source plant have not been assessed [38]. These studies on *P. angustifolia* contribute to the understanding of the drivers of invasion, however, empirical data on pollination, fruit and seed production, and soil seed bank viability is still lacking [11,29].

This study aimed to determine the drivers of *P. angustifolia* invasion in the high elevation grasslands of South Africa by investigating various aspects of its reproductive ecology.

## 2. Results

### 2.1. Visitation Frequencies of Floral Visitors

**Figure 1:**
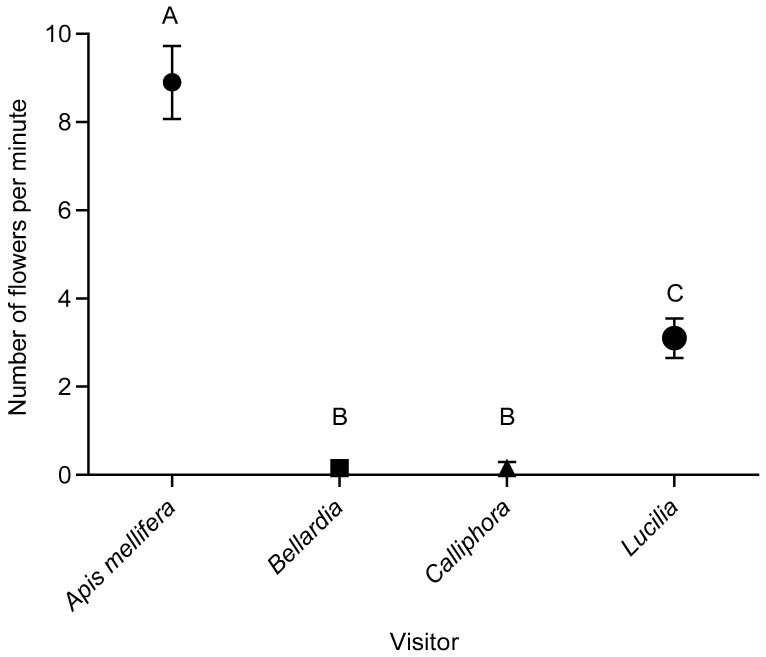
Number (mean ± SE) of flowers visited per minute by various insects on *Pyracantha angustifolia* in the grassland biome, Free State Province. Different letters indicate statistical significance (H-value = 62.68, *p* < 0.001, df = 3, Kruskal–Wallis).

One-minute observations showed that five insect species frequently visited *P. angustifolia* flowers (Figure 1). All insects observed touched the anther and were regarded as potential pollen dispersers with most of them searching for nectar. Analyses indicated significant differences in visitation frequencies among these species (H-value = 62.7, *p* < 0.001, df = 3). The highest mean ± SE visitation frequency was 8.9 ± 3.7 flowers min^−1^ (n = 20) for *Apis mellifera* L. (Apidae), followed by *Lucilia* sp. (Calliphoridae) with 3.1 ± 2.0 flowers Min^−1^ (n = 16), 0.2 ± 0.7 flowers Min^−1^ (n = 1) for *Calliphora* sp. (Calliphoridae) and *Bellardia* sp. (Calliphoridae), and 0.05 ± 0.2 flowers min^−1^ (n = 1) for an unidentified small bee species.

Five-minute observations (n = 20) totalling 400 min revealed 128 different individual insects visiting flowering branches. A mean ± SE of 1.3 ± 0.2 insects visited flowering branches per minute.

### 2.2. Pollen Loads

Fifty-seven individual insects comprising twelve different species from six different families were collected from *P. angustifolia* flowers (see Table S1). Diptera was the most diverse floral visitor group, and the largest number of species of any visiting insect family were from the Calliphoridae (Table S1). The largest number of insects caught visiting the flowers were *A. mellifera*, followed by *Calliphora* sp., and then *Lucilia*. *Eristalis* sp. carried the highest amount of *P. angustifolia* pollen (>1000 pollen grains) followed by *Eristalinus* sp., and then *Bellardia* sp.

*Bellardia* sp. carried the highest amount of pollen from other plant species (30.4 pollen grains), followed by *Eristalis* sp. (15.9), and then *Eristalinus* sp. (14.9). *Chrysomya*, *Spilostethus*, and *Syritta* species carried the lowest mean numbers of foreign pollen grains on their bodies (0.4, 0.7, and 1.2 pollen grains, respectively). All insects carried high proportions of *P. angustifolia* pollen (>70% purity) but *Chrysomya*, *Spilostethus*, *A. mellifera*, and *Lucilia* had the highest purity of 99.1, 97.5, 97.4, and 97.4%, respectively (see Supplementary Table S1). There were significant differences in the number of pollen grains only between *Apis mellifera* vs. *Calliphora* sp. and *A. mellifera* vs. *Lucilia* sp. (H-value = 15.3, *p* < 0.05, df = 4, Kruskal–Wallis). Species with a sample size of less than 2 (n = 1) were excluded from the analyses.

### 2.3. Pollinator Exclusions

There were no significant differences between the mean proportion of flowers yielding fruit between bagged and open flowering branches (*p* = 0.31, χ^2^ = 0.01), with open having a higher mean proportion of fruit yield (0.91 ± 2.4 SE, n = 44) compared to bagged branches (0.66 ± 5.1 SE, n = 8; see Supplementary Figure S2). In addition, all fruits sampled (n = 394) from both treatments produced the expected five seeds per fruit.

### 2.4. Shrub Size Distributions

The largest shrubs were located in the river site, followed by the open and then the rocky site (Figure S3). The most symmetric distribution was recorded in the open site (with k=2.96). A slightly more positive skewness was recorded in the river site (with k=2.46), while shrubs in rocky surroundings have the highest positive skewness (k=2.06). It is probably because the rocky surroundings provide some constraints on the ability of the shrubs to reach their full growth potential; while in an open environment, there are no constraints on growth. This is also evident when comparing the mean radius of the shrubs in the open terrains (with $\bar{R}=2.23\;m$) and rocky terrains (with $\bar{R}=1.78\;m$). Close to rivers, the shrubs were generally larger, with a mean radius $\bar{R}=2.63\;m$.

### 2.5. Fruit Estimates

**Figure 2:**
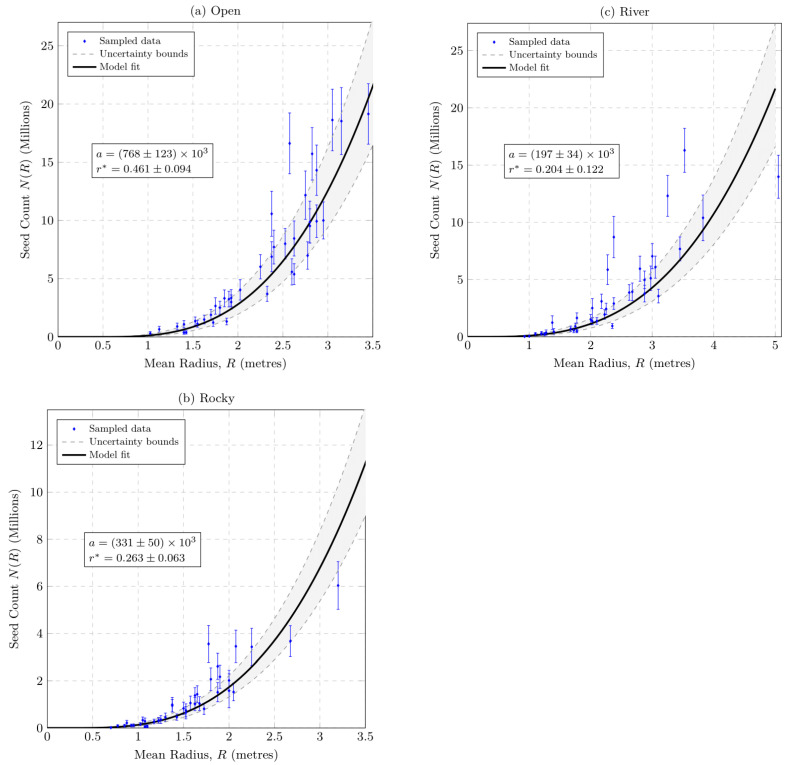
The mean radius of sampled *Pyracantha angustifolia* shrubs in relation to the mean number of seeds produced in millions for the three sampled field sites in (**a**) grassland, (**b**) rocky outcrops, and (**c**) riverine habitats. Dots on each graph show mean number of seeds and error bars indicate minimum and maximum seed counts.

Shrubs in open grassland produced more seeds compared to those in riverine and rocky outcrop sites. Each fruit produced a range of 3–6 seeds, with the majority (at least 70%) of fruits producing five seeds. There was an exponential increase in the number of seeds produced with an increase in plant radius (Figure 2). Small shrubs with a radius of 0.75 m already produced fruits. At least 47% of shrubs from the open grassland produced over five million seeds, while 25% and 2.5% of shrubs from river and rocky outcrop habitats, respectively, produced over five million seeds. Larger shrubs (>3 m radius) in open grassland can carry up to 20 million seeds per shrub. Shrubs in rocky and river environments produced fewer seeds (approximately less than 50% compared to open) but still numbered in the millions. This indicates that shrubs in open grassland produced more seeds than in other study areas. In open grassland, 55% of shrubs had a radius of over 2 m, with 12.5% and 60% of shrubs from rocky outcrop and river sites, respectively, having a radius of over 2 m (Table 1).

### 2.6. Pyracantha angustifolia Population Seed Counts

For each of the three sites (river, open, and rocky), seed count samples were recorded, as well as tree size and density in five 25 m^2^ quadrats. From the data it was possible to calculate the seed density, *n* (in millions of seeds per square meter of *P. angustifolia* invasion), for each of sites (Figure S4). Effectively, this is how many seeds are entering the environment per meter of invasion for each of the three sites. The probability distributions of seed density followed an exponential probability distribution function of the form

(1) $$P\left(n\right)=p_{E}\left(n;\lambda \right)=\frac{1}{n_{0}}e^{-\frac{n}{n_{0}}}.$$

The fit parameter, $n_{0}$, (in units of million seeds/m^2^), represents seed density for each of the sites. The seed densities per meter of invasion for open and rocky terrains are virtually identical with a characteristic seed density $n_{0}\;\approx \;1.7$ million seeds/m^2^, while close to rivers the characteristic density is considerably higher, with $n_{0}\;\approx \;3$ million seeds/m^2^ (Figure S4; Table 1).

The modelling of the *P. angustifolia* populations and seed productivity shows that the largest shrubs are currently found at the sampled river site. The larger shrubs also produced more seeds per meter of invasion. This was followed by the open grassland sites and finally by the rocky outcrops. However, it is worth noting that all sites sampled showed numbers of over 1 million seeds produced per meter of invasion (Table 1).

### 2.7. Seed Rain

High densities of loose seeds (mean ± SE 73.5 ± 11.3 seeds/m^2^) and seeds in whole fruits (3297 ± 282.1 seeds/m^2^) were found under *P. angustifolia* canopies (Table 2). Lower densities of both intact and loose seeds were found under *Rosa* (533.8 ± 522.1 seeds/m^2^ from intact fruits) and *L. sericea* canopies (12.5 ± 12.5 seeds/m^2^ from intact fruits). Seed traps under fence lines collected the smallest number of seeds (7 seeds in total) and no intact seeds. Only seed traps under *P. angustifolia* passed the D’Agostino and Pearson omnibus normality test, while *L. sericea* and *R. rubiginosa* samples were too small (n = 2 and n = 6, respectively). There were significant differences in total seed rain between *P. angustifolia* and *R. rubiginosa*, but no significant differences between *P. angustifolia* and *L. sericea*, and also between *R. rubiginosa* and *L. sericea* (H-value = 13.3, *p* < 0.05, df = 2). Fence lines were not included in the analyses because they had a sample size of one.

### 2.8. Soil Seed Bank

Soil cores from under and at the edges of the canopies of shrubs contained greater densities of intact or partially eaten fruits, whereas very few or no fruits and seeds were found at distances away from the shrubs (1, 2, 4, and 8 m from the canopy). There was a substantial decrease in mean density of seeds with increasing distance away from the centre of each shrub. The majority (>60%) of seeds found in the soil cores were decaying (easily broken when pressed between fingertips) and only viable seeds were kept in the analysis. The mean density of viable seeds was significantly greater in soil core samples from under shrubs than distances away from the centre. Significant differences in seed density were observed between the centre and soil samples from distances 1, 2, 4, and 8 m from the centre and between soil sample from the edge and 1, 2, 4, and 8 m away (H-value = 92.5, *p* < 0.05, df = 5, Kruskal–Wallis). There were no significant differences in seed density among soil samples from 1, 2, 4, and 8 m from the parent plant.

### 2.9. Seed Viability and Longevity in the Soil

There was high seed survival for the first three months after being buried; however, the seeds did not survive for longer than 6 months in the soil (Table 3). There were no differences between the two sites.

## 3. Discussion

All floral visitors observed in this study belonged to three insect orders, namely Hymenoptera, Diptera, and Hemiptera. The most frequent insect visitors to *P. angustifolia* flowers were *A. mellifera*. *Eristalinus* (Diptera) individuals carried the largest pollen loads, a high proportion of which was *P. angustifolia* pollen. As the species observed (especially *A. mellifera*) are typical generalist pollinators of many plant species in South Africa [46], this plant species is thus highly likely not to be pollen-limited outside of its native range. Nevertheless, visitation does not always equate to pollen deposition and successful reproduction, and a more in-depth study of the contribution of these insect visitors to seed set needs to be conducted [5].

All floral visitors had high *P. angustifolia* purity (>89%). In addition, floral visitors with high visitation rates also carried very pure pollen loads. *A. mellifera* had both the highest floral visitation frequency of 8.9 flowers min^−1^ and the highest pollen purity at 97.4%, followed by *Lucilia* sp. (3.1 flowers min^−1^; 97.4%). These results suggest that *A. mellifera* is the most important *P. angustifolia* floral visitor as it visits many flowers per minute, carries a relatively large amount of pollen, and the pollen purity is high. For the same reasons, *Lucilia* sp. Qualifies as the second most important pollinator, followed by both *Calliphora* and *Bellardia* although the latter two genera had the lowest visitation frequencies. Although *Chrysomya* sp. carried the purest (99.1%) pollen load, the species was not recorded in floral observation periods and therefore the study observations should be extended to cover as many visitors as possible. The high density of pollen found on visiting insects suggest the impact of *P. angustifolia* invasion and other IAPs on important crops and native plant pollination in the grasslands should be assessed in-depth [29]; (see [9] for grassland example).

The pollinator exclusion experiment proved that *P. angustifolia* can set fruit and produce seeds without floral visitors but further experimentation is needed with larger sample sizes to determine if they produce fruit by autonomous selfing. However, the results of this study strongly suggest that *P. angustifolia* is self-compatible. The ability of *P. angustifolia* to be self-compatible and/or use generalist pollinators implies that the species invasiveness is not dramatically hindered by lack of pollination vectors [47]. Therefore, it is expected that the generalist pollination behaviour allows increased probability of *P. angustifolia* spread [48,49].

Due to the high production of fruits per season produced by *P. angustifolia*, millions of seeds are available for wide distribution by dispersal agents [50]. One mature plant can produce more than 1 million seeds annually, translating to more than 1000 seeds per m^2^ [50]. In this study, it was shown that *P. angustifolia* produces an average of 2 million seeds per m^2^ of invaded area. The species produced double the number of seeds in open grassland compared with rocky outcrop grassland.

Shrubs in open grassland produced more seeds than shrubs in a rocky outcrop and riverine habitats in relation to shrub size but shrubs in open grassland and rocky outcrops produced similar numbers of seeds per given area in the field. However, shrubs produced at least 1.7 million seeds per square metre in all sites. Seed production in this study was higher than the one recorded by [50] where the shrubs produced 1000 seeds per square metre. High seed production plays a role in the probability of recruitment as it increases the chances of a seed reaching a favourable site for germination and seedling survival [51]. In addition, the large number of seeds produced increases the availability and attractiveness of fruits to frugivores [37,52,53]. Despite not lasting for a long period once on the ground, *P. angustifolia* fruits persist on the shrub through winter and summer seasons in large populations, and shrubs start fruiting while the previous season’s fruits are still on the shrub (pers. obs.). The observed persistence of fruits on shrubs implies that the fruits are available for frugivorous birds almost all year round (pers. obs), increasing the species’ invasiveness. As compared to other invaded systems, the shrubs also produce fruits “out of season” in Argentina [42]. Although information is lacking regarding reproductive ecology of the species in its native range [29], our results suggest the invasion hypothesis of evolution of increased competitive ability (EICA) due to increased seed or fruit production. The EICA states that after having been released from natural enemies, non-native species will allocate more energy in growth and/or reproduction (this re-allocation is due to genetic changes), which makes them more competitive [54]. The seeds ingested by birds were shown to be viable after being defecated and germination was improved by birds through removal of the fruit pulp [37].

The use of *P. Angustifolia* as perching sites for birds may also result in recruitment of other fleshy-fruited IAPs as birds might defecate them while perching on *P. angustifolia* branches [37,39,55]. This can facilitate the establishment of other species (potentially invasive species) within the invaded site as the thorny nature of the shrub protects developing seedlings growing close to the shrub from the harsh sunlight as well as grazing from livestock. High seed rain densities were observed under *P. angustifolia* canopies. High proportions of seed from intact fruits under *P. angustifolia* canopies indicates that many seeds reached the ground in intact fruits, also confirmed by the soil seed bank analysis. The presence of *P. angustifolia* loose seed under the fence, *R. rubiginosa* and *L. sericea*, reveals that birds using the latter as perching structures [38] also use *P. angustifolia*. Although Chari et al. [29] mentioned seed dispersal by animals, human translocation, and water and wind dispersal should also be recorded. Unexpectedly, there were whole fruits collected from seed traps under *R. rubiginosa* and *L. sericea* canopies. These fruits might have been dispersed by the wind to these understoreys as *P. angustifolia* fruits can also be wind dispersed [29].

The decrease in the soil seed bank density with an increase in distance away from the parent plant has also been revealed by other studies [56,57]. A high proportion of seeds crumbled under light pressure when handled, suggesting that they were not viable and susceptible to decomposition. Although the seeds have a short life span in the soil (<6 months), many seeds should find suitable sites for germination through bird dispersal soon after maturing. Our results show that the species must depend more on bird dispersal for its spread away from the parent population rather than wind or water dispersal in grassland habitats.

Gioria et al. [58] tested EICA in terms of the invasiveness hypothesis, which predicted that the ability of an IAP to naturalise in an invaded area can be seen by species forming persistent seed banks. Our results revealed *P. angustifolia* to be an exception to this hypothesis as seeds only survived less than six months in the soil after burial. This is unlike some aggressive invaders such as *Rubus alceifolius* (Rosaceae) that produces seeds that persist in the soil for much longer periods, i.e., for at least 5 years [59] Marks 1983. Although the seeds lack longevity in the soil, they can germinate across a wide range of environmental conditions in terms of soil chemical, temperature, and moisture levels [29,36]. They also readily start germinating a month after dispersal, with only 7% of total seeds planted as whole fruits germinating, as revealed by germination experiments of ingested and manually depulped seeds [37]. The chances of *P. angustifolia* re-establishment through the soil seed bank post-clearing are small because of the seeds have low survival rates in the soil and need pulp removal for faster germination [60].

*Pyracantha angustifolia* is a formidable invader due to its ability to self-pollinate and use generalist insect pollinators, high seed production, high seed viability straight after dispersal, having various seed dispersal agents including birds, water, wind and other animals, and having large thorns making the plant difficult to manage manually. Also, the short life span of seeds may prevent excessive recolonization after clearing invaded sites, and thus management can be implemented perhaps with manual clearing, 6-month and annual monitoring up to three years, and potentially long-term plans for biocontrol investigations into agents targeting reproductive structures to control larger invasions that are too costly to manually remove.

## 4. Materials and Methods

### 4.1. Study Area

**Figure 3:**
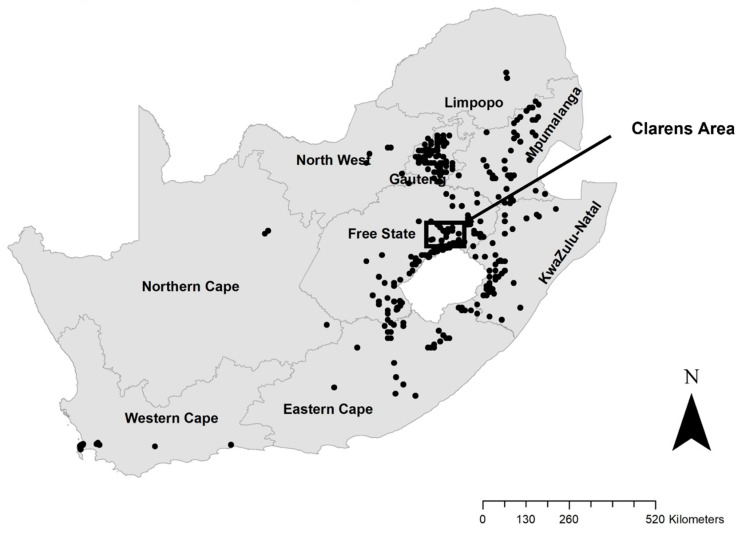
*Pyracantha angustifolia* distribution in South Africa. The black square indicates the study site. Locality data sourced from GBIF [25] Occurrence Download, viewed 26 January 2021, from https://doi.org/10.15468/dl.h3hp2c (accessed on 26 January 2021) and Southern African Plant Invasion Atlas (SAPIA) database (Adapted with permission [30]).

The data were collected in the eastern Free State Province of South Africa (Figure 3) from April 2018 to June 2020. The study area is typified by private farmland with patches of natural grassland, rocky outcrops, and sandstone cliffs deeply incised by rivers [61]. The area receives an average rainfall of 600 to 1000 mm per annum, and occasional snow and frequent frost in winter. The annual average minimum and maximum temperatures are 6 °C and 26 °C, respectively, although frost usually reduces the temperature to well below freezing in winter [62]. The area falls under the grassland biome (Mesic Highveld Grassland) [61]. More specifically; the Eastern Free State Clay Grassland and Eastern Free State Sandy Grassland, which are now mostly transformed by the agricultural industry [61].

The study area has been invaded by a number woody species including *Rosa rubiginosa* L., *Cotoneaster* Medik spp., *Rubus* L. spp., (all Rosaceae), *Robinia pseudoacacia* L., *Gleditsia traicanthos* L. (both Fabaceae) and *Salix* L., nom. cons. spp. (Salicaceae) [63].

*Pyracantha angustifolia*, native to southwest China, was introduced to South Africa as an ornamental plant, for security, hedging, and potentially as a source of vitamin C [29]. In South Africa, *Pyracantha angustifolia* invades high altitude grasslands, bush clumps, erosion channels, forests, rocky ridges, and watercourses [29]. The thorny plant forms dense monocultures and competes with native grassland plant species, thereby reducing grazing capacity of the grassland and changing the ecosystem [12]. The species’ fruiting period ranges between April and November but mature fruits may remain on the shrub for the majority of the year ([29], Adams pers. obs). Leaves are alternatively arranged, dull dark green above, and greyish underneath [12]. The leaves have a diagnostic notched tip. Flowers are arranged in compound corymbs of 2 to 4 cm in diameter that can contain from a few to 30 flowers [29]. These flowers each have five white petals (5 to 12 mm across), five small sepals, and twenty stamens. Mature fruits become either orange-red or orange-yellow with age [64]. All *Pyracantha* species have somewhat similar flowers and fruits but can be distinguished using leaf morphology [65].

### 4.2. Visitation Frequencies of Floral Visitors

Data on floral visitors and pollinator exclusion experiments were collected in December 2018 near Clarens Town (28°32′8″ S; 28°25′2″ E) from flowering *P. angustifolia* shrubs. The observations were performed on sunny days between 09:00 and 13:00. To investigate the visitation rates of insect visitors to flowers of *P. angustifolia*, timed observations of visitors were conducted, in which individual insect visitors were observed for one minute and the number of flowers visited was noted. Eighty observations were randomly performed on at least ten different shrubs and included insects of different species. Visitation frequencies were expressed as the number of flowers visited per minute. Data normality was tested using D’Agostino and Pearson omnibus normality tests. As the data collected were not normally distributed a non-parametric Kruskal–Wallis test was used to determine significant differences (*p* < 0.05) in mean visitation frequencies between species of insect visitors. Data were analysed using GraphPad Prism 5 statistical software [66]. Insects were identified to genus level if possible; otherwise, morpho-species level identifications were used.

To determine the visitation frequency of floral visitors, flowering branches were observed for five minutes and the number of all visitors was noted and counted. Flowering branches were randomly chosen, and branch size was not taken into consideration as the interest was on flowers. A total of twenty branches were consecutively observed for five minutes each, totalling 100 min of observation over two days. Visitation frequencies for floral visitors were calculated as the average number of individual insects observed visiting per minute. Insects were not identified to species, only the number of insect individuals visiting the flowers were counted.

### 4.3. Pollen Loads

Pollen load, referring to the amount and type of pollen carried by a floral visitor, was determined to assess the amount of *P. angustifolia* pollen transferred to visiting insects and if these insects had visited other plant species or were showing constancy (i.e., only visiting *P. angustifolia*). To examine the pollen load on insects visiting *P. angustifolia* flowers, insects visiting flowers were caught with an insect net and placed in 5 mL microcentrifuge tubes, during the flowering period of December 2018. Insects were dabbed with fuchsin gel [67] to collect pollen deposited on the insects’ bodies. Microscope slides were made by melting the fuchsin gel and covering the melted gel with a glass cover slip. The slides were heated with a lighter at a distance of at least 5 cm. Pollen grains were counted and identified to genus level (where possible) under a light microscope at 40× magnification. All insects were pinned for identification and reference (see Supplementary Information, Figure S1).

Total *P. angustifolia* pollen was counted on each slide and the mean number of pollen grains per insect species was calculated. Pollen grains from other plant species were similarly identified and counted. Mean pollen number with standard error was determined for insect species for which more than one individual was caught. The exact value of pollen grains is presented for insect species for which only one individual was caught. Insect species were identified to genus or species level where possible. The mean numbers of *P. angustifolia* pollen grains between insect species were compared using Kruskal–Wallis statistics followed by Dunn’s multiple comparison test on GraphPad Prism 5 for any insect species for which more than one individual had been caught. The mean number of foreign pollen grains (pollen from other plant species flowering sympatrically) carried by each insect were also determined for each insect species. Percentage *P. angustifolia* pollen purity (*Pp*) (how pure the pollen collected from insects was) was determined using the following formula:

(2) $$Pp=\frac{\mathrm{Total}\;Pyracantha\;angustifolia\;\mathrm{pollen}}{\mathrm{Total}\;Pyracantha\;angustifolia\;\mathrm{pollen}+\mathrm{total}\;\mathrm{other}\;\mathrm{specie}s^{\prime }\mathrm{pollen}}\times 100\%.$$

### 4.4. Pollinator Exclusions

To determine the natural fruit set and if pollinators are needed for sexual reproduction in *P. angustifolia*, a total of eight 30 cm branches on four randomly selected shrubs growing in the open grassland were bagged with fine nylon mesh to exclude all floral visitors. The branches were bagged before any buds had opened. This tested for autogamous pollination and seed set. The bags were not removed until fruits were collected to avoid fruits falling on the ground or being eaten by birds. The number of buds at the start of the experiment were counted and the point to which these were counted along the branch was marked with fluorescent tape.

Similarly, for investigating natural fruit set from open pollination and to compare natural fruit set to bagged branches, the number of flowers were noted for each of the 44 flowering branch tips and marked with a plastic band. The flowers were left open to pollinators (unbagged). After flowers had senesced and during the early development of the fruits, branches were bagged to avoid fruits falling off or being eaten by birds.

Fruit set on bagged and open branches was compared using a generalised estimating equation (GEE) in IBM SSPS Statistics version 25 software. The GEE was used because it allows one to account for plant effects and differences in flower numbers. Plant was used as the subject variable. The model followed a binomial distribution transformed with a logit link function and employed an exchangeable correlation matrix. In addition, ten fruits per branch were cut open and the number of seeds inside were counted to determine if fruits contained seeds and how many were produced per fruit on average. A chi-square test was also used to test for the null hypothesis, i.e., that there were no differences in seed set between bagged and non-bagged branches.

### 4.5. Fruit Estimates

In order to determine fruit production, and therefore number of viable seeds produced by *P. angustifolia* populations invading different habitats in South Africa, representative shrubs and populations were selected. Roadside surveys and the literature suggested open grasslands, rocky hillsides, and a riverine habitat as key regions for invasion and representative sites were accordingly selected [10,15]. All three sites were on agricultural land, however, *P. angustifolia* stands were not in the cultivated areas but in the adjacent natural areas with low disturbance. At each site, 40 shrubs of different size classes were randomly selected from the population sampled. Fruit production was estimated towards the end of the fruiting season (August to September 2019).

**Figure 4:**
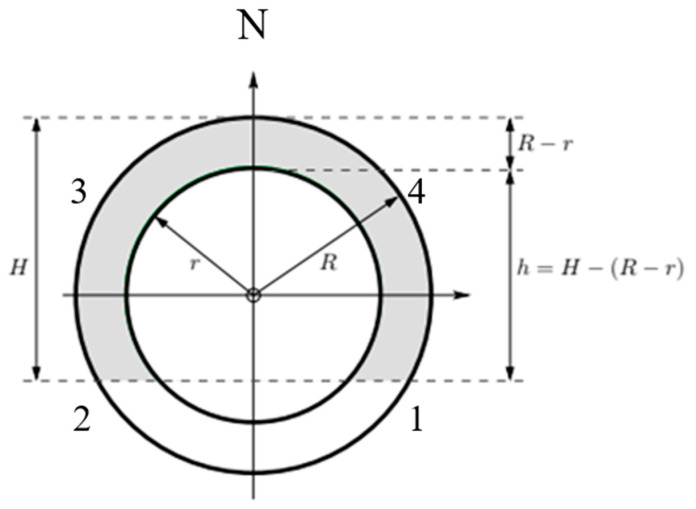
Shrub’s cross-sectional view assuming an approximate spherical shape of the shrub indicating the effective fruiting distance, *R* − *r*, (shaded) and fruiting height, *h*). The numbers around the shrub indicate the quadrants (numbers 1 to 4). *H* refers to the fruiting height of the plant.

To investigate the number of seeds produced per annum and thus give an indication of the productivity of populations, we investigated the relationship between plant volume and the number of seeds. Due to the high number of fruits produced per shrub, it was necessary to sub-sample fruits and seeds per shrub. The following method was used to estimate the number of fruits per shrub. Firstly, the size of the shrub on which the number of fruits was to be calculated was measured. This was achieved by measuring plant height (ground level to the highest leaves) and two diameters at the widest points of the shrub in two directions: one in a north to south direction and one east to west. Shrubs were divided into quadrants (southwest, northwest, northeast, and southeast). The fruiting distance within each quadrant was then determined by placing a measuring pole into each quadrant towards the centre of the shrub and measuring the distance from the outermost fruits to the innermost fruits. Following this, the number of fruits per quadrant was estimated by randomly placing a wire cube in the fruiting area and counting fruits inside the cube (10 × 10 × 10 cm = 1000 cm^3^ = 0.001 m^3^). Five random cube samples were taken per quadrant. The total number of fruits produced per shrub was then extrapolated (see Statistics section below, Figure 4).

In determining the average number of seeds per fruit, 10 fruits were collected in each quadrant per shrub and stored in paper bags. In the laboratory, the fruit flesh was removed, and the seeds inside were counted and noted. The mean (±SE) number of seeds produced per fruit was then used to calculate the number of seeds produced per sampled shrub using the extrapolated total number of fruits per shrub. In order to determine the average shrub density and the plant demography at each of the sites (rocky, river, and open) five randomly placed 5 × 5 m quadrats were selected at each site. Within each quadrat, all shrubs found growing in the quadrat were identified and diameter, height and fruiting distance were measured. Unlike other woody invasive plants (e.g., [68], basal stem circumference was not measured due to the multi-stemmed nature of the species and large thorns making access impossible. The shrub size distributions showed a tendency to be skewed to the right, therefore a Weibull distribution function was used to fit to the results

(3) $$P\left(R\right)=p_{W}\left(R;\lambda ,\;k\right)=\frac{k}{\lambda }\;{\left(\frac{R}{\lambda }\right)}^{k-1}e^{-{\left(\frac{R}{\lambda }\right)}^{k}}.$$

Here, k is a shape parameter for the distribution, determining skewness of the distribution, while λ is a scale parameter (in units of meters). The skewness of the distributions provides some indication of the impact of the nature of the surroundings on the growth of *Pyracantha angustifolia*.

The following section provides details on the mathematical model used in calculating the fruit estimates.

### 4.6. Seed Count Model

In order to determine the total seed count for the shrubs at each site, the following model was developed by (a) using the seed count samples in the various quadrants to estimate the total seed count for the shrub, and then (b) establishing a functional relationship between the total seed count and the plant radius. This was performed for each of the sites to establish any differences between the seed counts at each site.

(a) Determining seed counts.

It is assumed that the shrubs are spherically shaped with a total outer radius *R*, with the seeds located within a shell with an inner radius, *r*, and in a fruiting height of the shrub, *H*, as shown in Figure 4.

Using the formula for the volume of a segment of a sphere of radius, *r*, and segment height, *h*,

(4) $$V=\frac{1}{3}\pi h^{2}\left(3r-h\right),$$

one obtains the volume of a segment of a spherical shell by subtracting the inner segment from the outer segment:

(5) $$V\left(R,r,H\right)=\frac{1}{3}\pi H^{2}\left(3R-H\right)-\frac{1}{3}\pi h^{2}\left(3r-h\right).$$

Using the fruiting height

(6) $$h=H-\left(R-r\right)$$

as shown in Figure 4, the volume simplifies to

(7) $$V\left(R,r,H\right)=\frac{1}{3}\pi H^{2}\left(3R-H\right)-\frac{1}{3}\pi h^{2}\left(3r-h\right).$$

From the sampling process for each shrub discussed in the previous section, the quadrant seed count, *n_q_* (*q* = 1, 2, 3, 4), was obtained for each quadrant based on 5 samples per quadrant. Using this quadrant seed count, the quadrant seed density, *ρ_q_*, could then be estimated as

(8) $$\rho_{q}=5\;n_{q}\times 1000$$

where the factor of 1000 is used to convert to m^3^ (since, as previously noted, each sample container had a volume of 0.001 m^3^). Using the shrub height, *H*, and the average radius, *R*, (obtained from the diameter measurements), the fruiting distance for each quadrant, $r_{q}$, the volume of each quadrant, $V_{q}$, was calculated using equation

(9) $$V_{q}=\frac{1}{4}V\left(R,r_{q},H\right).$$

Based on the quadrant seed density and quadrant volume, the estimated total seed count, $\hat{N}$, for the shrub can be calculated by summing over all the quadrants:

(10) $$\hat{N}=\overset{4}{\underset{q=1}{\sum }}\rho_{q}\;V_{q}.$$

(b) Functional relationship.

We assume that the shrub radius is a proxy for age, so that for a shrub radius, *R*, less than some value $r^{*}$, the shrub will not produce any seeds. Only when the size is $R>r^{*}$ (i.e., the plant has reached a certain age) will the shrub produce seeds. From that point, the number of seeds must scale as the volume, i.e., $N\;\sim {\left(R-r^{*}\right)}^{3}$. The model used to relate the number of seeds to the shrub radius is therefore

(11) $$N\left(R\right)=\left\{\begin{array}{r} 0,\;\;for\;R<r^{*}\\ a{\left(R-r^{*}\right)}^{3},\;\;for\;R\geq r^{*} \end{array}.\right.$$

Here *a* is a parameter that is used to scale the “volume”-to-seed count. Furthermore, note that only sexually mature shrubs were included in the study.

### 4.7. Seed Rain

Seed rain below the shrubs was calculated per shrub. This was only conducted in the open grassland as the environmental aspects of the other two sites precluded them from the experiment but it is assumed that the rain would be similar for all sites. Seed traps (n = 55), comprising shallow perforated plastic containers (diameter 30 cm × 15 cm deep) covered with wire netting to exclude rodents and birds, were secured to the ground with metal pegs beneath shrub canopies to estimate the density of seed arriving on that surface. Two traps were placed under mature *P. angustifolia* at the site. In addition, the same seed traps were placed randomly under surrounding shrubs (*R. rubiginosa* and *Leucosidea sericea* Eckl. & Zeyh, (Rosaceae) as well as under nearby fence lines. It was assumed that *P. angustifolia* seed found in seed traps under other shrub species and fence lines had been dispersed there by birds or mammals. Seed traps were emptied monthly, seed was cleaned and sorted, and the number of seeds arriving in each trap were counted. For whole fruits emptied from the traps, the number of fruits was multiplied by the average number of seeds to calculate the total number of seeds in the trap. Forty-four seed traps, two per shrub, were placed under *P. angustifolia*, one trap per shrub placed under two *L. sericea* shrubs, six traps under *R. rubiginosa* shrubs, and one under fence lines. The total amount of seed traps amounts to 53 seed traps. Seed traps were placed in the field for 12 months to cover all the seasons (October 2018 to September 2019).

Two perpendicular shrub diameters were measured, and canopy area was calculated. Seed rain (SR) was calculated using the following formula [38]:

SR = (SF/TA) × CA.(12)

Seed fall (SF) refers to the number of seeds counted in the seed trap and total area (TA) refers to the sum of seed traps area. Canopy area (CA) was the shrub canopy area calculated using the following formula:

CA = (Pi/4) × canopy1 × canopy2(13)

where canopy1 and canopy2 were the two perpendicular diameters of the shrubs.

Ultimately, mean annual seed rain (mean ± SE) expressed as the number of seeds per square metre was determined for loose seeds, seeds in intact fruits, and total seed number for seed traps under different canopies and fence lines. Statistically significant differences were also determined using Kruskal–Wallis tests followed by Dunn’s multiple comparison tests using GraphPad Prism 5. Data were checked for normality via D’Agostino and Pearson omnibus normality test before the analyses.

### 4.8. Soil Seed Bank

To determine the seed density in the soil around the source plant, soil cores were taken during the fruiting season of *P. angustifolia* (August 2018). Soil cores were taken from 20 randomly selected sexually mature shrubs, 2–3 m in height, that had fruits on them and were at least 16 m away from another *P. angustifolia* plants so as not to sample fruits fallen from neighbouring shrubs. Six soil samples (using a soil auger, 7.5 cm diameter × 20 cm depth) were taken in one random direction away from each of the 20 focal plants, thus 120 soil core samples were taken.

The first sample was taken directly under the shrub canopy, the second one at the edge of the canopy, and the remaining four samples at 1 m, 2 m, 4 m, and 8 m from the canopy edge. Soil samples were collected for seed bank assessments using the modified methods of Holmes [69]. Soil samples were passed through a sieve (1 mm) and fine particles were removed with only seeds and large particles remaining in the sieve. The sieved *P. angustifolia* seeds could be clearly distinguished from soil clumps. Seeds collected from the field were stored in the laboratory in paper bags until counted. The average number of seeds per area was calculated. Intact seeds were assumed to be viable.

### 4.9. Seed Viability and Longevity in the Soil

Fresh fruits were harvested from more than 20 randomly selected *P. angustifolia* shrubs growing in the eastern Free State. The flesh was removed from the seeds. Seeds are best cleaned while fresh because it is difficult to remove the fleshy material once dry. Seeds were stored in a cool, dry, dark container for two months. Seeds of many Rosaceae species exhibit double dormancy due to their hard, impermeable seed coats and the physiological condition of their embryos. Because of the dormancy in these seeds, the International Seed Testing Association recommends the use of tetrazolium staining rather than germination tests for evaluation of seed quality [70]. Seeds were stained by first soaking them in water for 18 h, then the distal (furthest away from the seed embryo) third of the seeds was removed with a transverse cut, and the seeds were then placed in a 1.0% solution of tetrazolium chloride for 20 to 24 h. Viable seeds usually stain completely, but seeds are considered viable if only the radicle tip and the distal third of the cotyledons are unstained [70].

Before the seeds were buried, a sub-sample of 400 seeds was tested for viability, all were viable. The seeds were then divided into groups of 100 seeds and placed in 10 cm × 5 cm individual perforated plastic mesh bags (garden shade cloth). Seeds were then buried at two sites near the town of Clarens in the Free State Province between June 2020 and January 2021. At each site, four 50 cm deep and 7.5 cm wide holes were dug. Each hole was at least five meters away from the next one. In each hole, 12 seed bags were placed and covered with soil. The location of each hole was marked with a 30 cm metal stake protruding from the ground. The sites were as follows: Site 1, grassland (−28.542771°; 28.507778°), was in a valley bottom; valley facing north; loam to clay soils. Site 2 was in a valley thicket (−28.543331°; 28.506207°); rocky outcrop, west-facing slope; sand, shale soils. At each site two replicates were conducted. Each replicate included 12 perforated bags each containing 100 seeds per bag. Bags were removed every 3 months after which seeds were tested for viability as described above.

## Figures and Tables

**Table 1:** Summary of the number of seeds per invasion site and shrub size for the three different terrains sampled in the study.

	Seed Density (Million Seeds/m^2^)	Mean Shrub Radius (m)	Notes
River	3.022	2.63	High seed number. Larger shrubs.
Open	1.738	2.23	Seeds more dispersed, large and small shrubs.
Rocky	1.653	1.78	Seeds more dispersed. Smaller shrubs.

**Table 2:** *Pyracantha angustifolia* seed rain in seeds per square metre (mean ± SE) under *P. angustifolia*, *Rosa rubiginosa, Leucosidea sericea,* and fence lines. Densities of intact and loose seeds were analysed separately.

	*P. angustifolia* (n = 44)	*L. sericea* (n = 2)	*R. rubiginosa* (n = 6)	Fence (n = 1)	F Value	*p*-Value
Total seed rain	56,766 ± 7735	71 ± 55	2310 ± 2244	7	6.1	0.0054
Seed rain from whole fruits	55,395 ± 7490	61 ± 61	2248 ± 3195	0	12.8	0.0001
Loose seed rain	1371 ± 331	10 ± 6	62 ± 50	7	12.5	0.0329

**Table 3:** Percentage (mean ± SE) survival of *Pyracantha angustifolia* seeds after being buried in the soil for up to six months at two field sites.

	Seed Survival (%)		
Months in the Soil	1	3	6
Site 1	98.0 ± 0.0	94.3 ± 0.7	0
Site 2	98.0 ± 0.0	90.6 ± 1.4	0

## Data Availability

The data presented in this study are available on request from the corresponding author.

## Supplementary Materials

The following supporting information can be downloaded at: https://www.mdpi.com/article/10.3390/plants12061308/s1, Figure S1: Process followed to collect pollen grains from insects visiting Pyracantha angustifolia flowers. The insects visiting flowers were caught with an insect net and placed in 5 mL microcentrifuge tubes (A). Insects were dabbed with Fuchsin gel (B) to collect pollen deposited on the insect’s body. Microscope slides were made by melting the Fuchsin gel (C) and covering the melted gel with a glass cover slip (D). Pollen grains were counted and identified to genus level (where possible) under a light microscope at 40× magnification (E) and insects were pinned for identification to genus level (F); Figure S2: Back-transformed mean (± SE) proportion of seed set per branch tip from bagged and open flower buds. There were no statistically significant differences (*p* > 0.05) in the proportion of fruit set between bagged and open branches; Figure S3: The probability distribution of Pyracantha angustifolia radius for shrubs in three field sites, (a) Open grassland, (b) Rocky outcrop and (c) River habitats; Figure S4: The probability distribution of seed count density (million seeds/m^2^) from three field sites in (a) Open grassland, (b) Rocky outcrop and (c) River habitats for seed production by Pyracantha angustifolia; Table S1: Number of pollen grains found on each floral visiting insect collected from flowering Pyracantha angustifolia shrubs during the flowering period (December 2018). There were significant differences on number of pollen grains only between *Apis mellifera* vs. *Calliphora* sp. and *A. mellifera* vs. *Lucilia* sp. (H-value = 15.3, *p* < 0.05, df = 4, Kruskal–Wallis). Species with sample size of less than 2 (n = 1) were excluded from the analyses.
