# Supplementary material for: Reproductive Ecology of the Invasive Alien Shrub Pyracantha angustifolia in the Grassland Biome, South Africa
Source: Plants (Basel). 2023 Mar 14;12(6):1308. doi: 10.3390/plants12061308 (PMC10058135; doi:10.3390/plants12061308)
Supplement: Supplementary file 1 [file plants-12-01308-s001.zip › plants-2198661-supplementary.pdf]

SUPPLEMENTARY MATERIAL

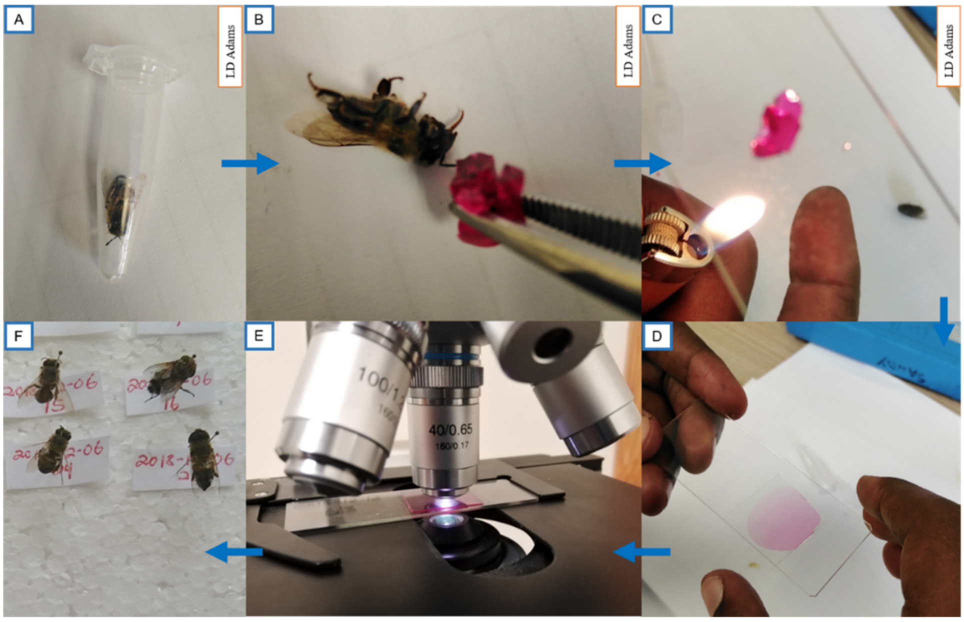

Figure S1. Process followed to collect pollen grains from insects visiting *P. angustifolia* flowers. The insects visiting flowers were caught with an insect net and placed in 5 ml microcentrifuge tubes (A). Insects were dabbed with Fuchsin gel (B) to collect pollen deposited on the insect's body. Microscope slides were made by melting the Fuchsin gel (C) and covering the melted gel with a glass cover slip (D). Pollen grains were counted and identified to genus level (where possible) under a light microscope at 40x magnification (E) and insects were pinned for identification to genus level (F).

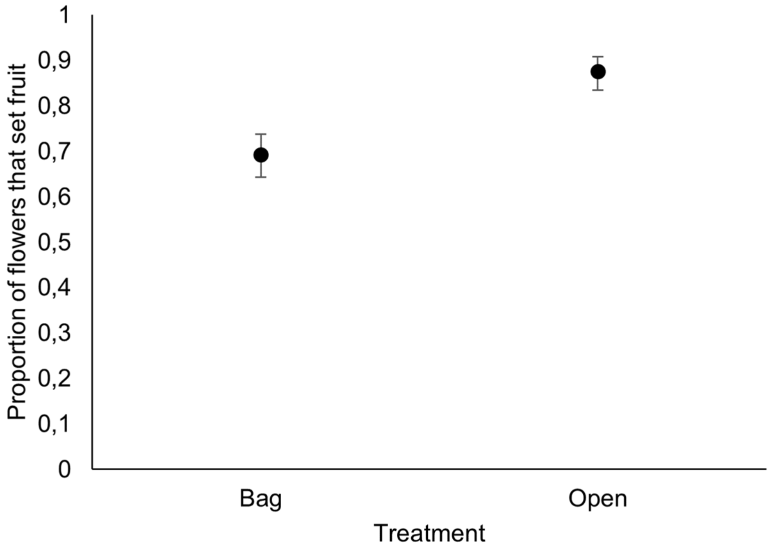

Figure S2. Back-transformed mean (± SE) proportion of seed set per branch tip from bagged and open flower buds. There were no statistically significant differences (P>0.05) in the proportion of fruit set between bagged and open branches.

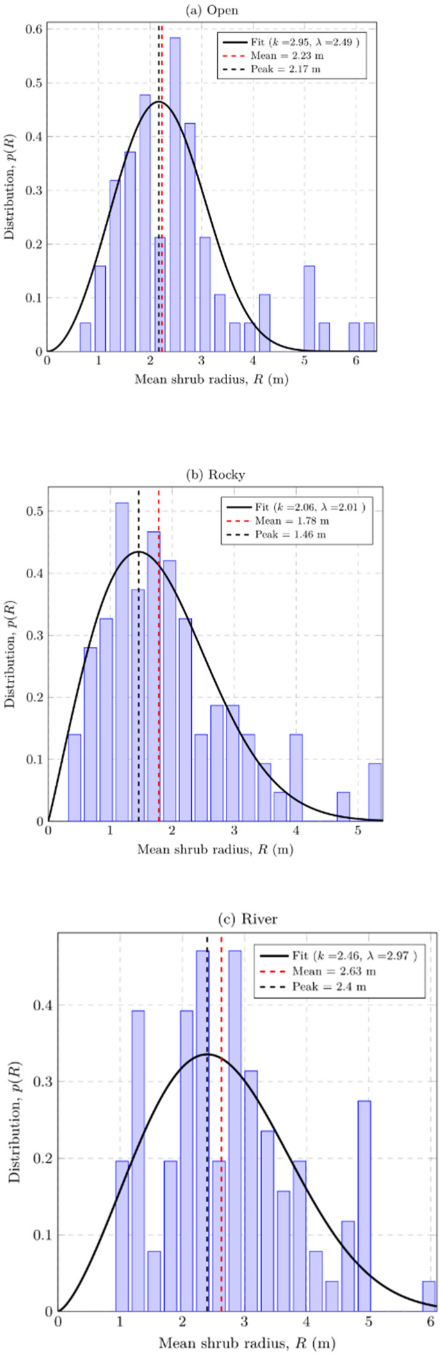

Figure S3. The probability distribution of *Pyracantha angustifolia* radius for shrubs in three field sites, (a) Open grassland, (b) Rocky outcrop and (c) River habitats.

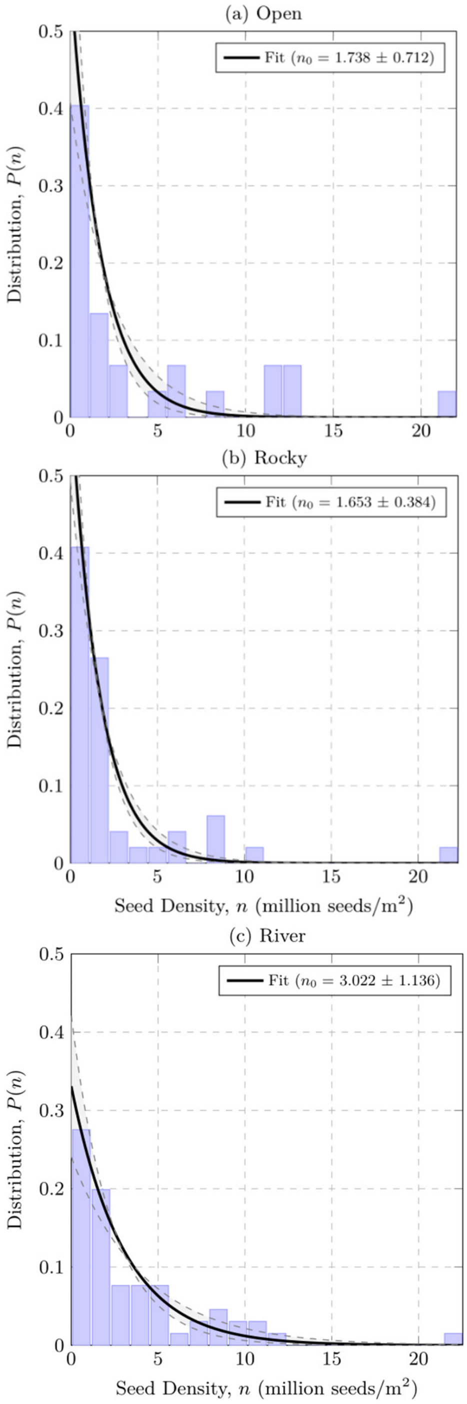

Figure S4. The probability distribution of seed count density (million seeds/m^2) from three field sites in (a) Open grassland, (b) Rocky outcrop and (c) River habitats for seed production by *Pyracantha angustifolia*.

Table S1 Number of pollen grains found on each floral visiting insect collected from flowering *Pyracantha angustifolia* shrubs during the flowering period (December 2018). There were significant differences on number of pollen grains only between *Apis mellifera* vs *Calliphora* sp. and *A. mellifera* vs *Lucilia* sp (H-value = 15.3, $P < 0.05$, df = 4, Kruskal-Wallis). Species with sample size of less than 2 (n = 1) were excluded from the analyses.

Order	Family	Subfamily	Species	Mean± SE pollen grains	Mean Other pollen grains	*Pyracantha angustifolia* pollen purity (*Pp* in %)
Hymenoptera	Apidae	Apinae	*Apis mellifera* (n=24)	333.6 ± 71.1	8.9	97.4
Diptera	Calliphoridae	Calliphorinae	*Calliphora* sp. 1 (n=11)	70.2 ± 27.0	3.2	95.7
			Chrysomya sp. 1 (n=2)	49.0 ± 34.0	0.4	99.1
		Luciliinae	*Lucilia* sp. 1 (n=9)	85.9 ±44.2	2.3	97.4
		Polleniinae	*Pollenia* sp. 1 (n=1)	68.0	5.6	92.4
			Bellardia sp. 1 (n=1)	370.0	30.4	92.4
	Syrphidae	Eristalinae	*Eristalinus* sp. 1 (n=4)	467.8 ± 253.9	14.9	96.9
			Eristalis sp. 1 (n=1)	>1 000.0	15.9	98.4
			Syritta sp. 1 (n=1)	10.0	1.2	89.1
	Asilidae		*Dysmachus* sp. 1 (n=1)	7.0	2.7	72.4
	Sarcophagidae		*Sarcophaga* sp. 1 (n=1)	32.0	1.6	95.4
Hemiptera	Lygaeidae		*Spilostethus* sp. 1 (n=1)	26.0	0.7	97.5
